# Complex regulatory role of DNA methylation in caste- and age-specific expression of a termite

**DOI:** 10.1098/rsob.220047

**Published:** 2022-07-06

**Authors:** Mark C. Harrison, Elias Dohmen, Simon George, David Sillam-Dussès, Sarah Séité, Mireille Vasseur-Cognet

**Affiliations:** ^1^ Institute for Evolution and Biodiversity, University of Münster, Münster, Germany; ^2^ Biocampus, CNRS, INSERM, Montpellier, France; ^3^ University Sorbonne Paris Nord, Laboratory of Experimental and Comparative Ethology (LEEC), UR4443, Villetaneuse, France; ^4^ UMR IRD 242, UPEC, CNRS 7618, UPMC 113, INRAE 1392, Institute of Ecology and Environmental Sciences of Paris, Paris 7 113, Bondy, France; ^5^ University of Paris-Est, Créteil, France; ^6^ INSERM, Paris, France

**Keywords:** DNA methylation, termites, caste-biased expression, alternative splicing, ageing, fertility

## Abstract

The reproductive castes of eusocial insects are often characterized by extreme lifespans and reproductive output, indicating an absence of the fecundity/longevity trade-off. The role of DNA methylation in the regulation of caste- and age-specific gene expression in eusocial insects is controversial. While some studies find a clear link to caste formation in honeybees and ants, others find no correlation when replication is increased across independent colonies. Although recent studies have identified transcription patterns involved in the maintenance of high reproduction throughout the long lives of queens, the role of DNA methylation in the regulation of these genes is unknown. We carried out a comparative analysis of DNA methylation in the regulation of caste-specific transcription and its importance for the regulation of fertility and longevity in queens of the higher termite *Macrotermes natalensis*. We found evidence for significant, well-regulated changes in DNA methylation in mature compared to young queens, especially in several genes related to ageing and fecundity in mature queens. We also found a strong link between methylation and caste-specific alternative splicing. This study reveals a complex regulatory role of fat body DNA methylation both in the division of labour in termites, and during the reproductive maturation of queens.

## Graphical abstract

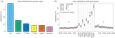

## 1. Introduction

DNA methylation, the epigenetic modification of DNA, is widespread among eukaryotes and is known to be important for transcriptional regulation of genes and repression of transposable elements (TEs) [1]. Age-related changes in DNA methylation levels and an increased variability known as epigenetic drift have been recognized as an important hallmark of ageing in mammals [2,3]. DNA methylation has garnered considerable attention within social insects, with an apparent role in the regulation of sterile and fertile castes in honeybees [4] and in ants [5]. A more recent study found a significant role of methylation in the task division of worker bees [6]. However, there remains considerable debate surrounding the universality of the role of DNA methylation in the transcriptional regulation of caste-specific genes in eusocial insects [7–9]. In bumblebees, DNA methylation appears to be more important for worker reproduction [10] than for caste differentiation [11]. Two studies found no influence of DNA methylation on the formation of behavioural castes in a wasp [9] and an ant [8,9] that live in simple societies. In fact, the authors of the latter study claimed previous evidence for the role of DNA methylation in the division of labour was weak and that further studies required more robust methodology, especially greater replication [8]. All of these studies measured DNA methylation in brains, in order to detect regulatory patterns related to behavioural differences, or analysed whole-body methylation often due to difficulties in obtaining sufficient DNA.

Most of these studies have concentrated on social Hymenoptera (ants, bees and wasps), with the exception of two studies on the role of DNA methylation in the division of labour in adult termites. The first of these studies investigated whole-body methylation patterns for the lower, drywood termite *Zootermopsis nevadensis* [12], which forms simple colonies, in which workers retain the possibility to become fertile [13]. In the second study, head methylomes of the subterranean termite *Reticulitermes speratus* were investigated, a species with an intermediate level of social complexity [14]. While the first study found large differences between castes in *Z. nevadensis* [12], Shigenobu *et al.* [14] found very strong correlations in DNA methylation patterns between castes of *R. speratus*. However, in the first study, limited replication was performed within one single colony, while in the second study, non-replicated castes were sampled from different colonies, so that the effect of colony-specific variation, inherent in previous studies [8], could not be excluded in either of these studies. The general role of DNA methylation in the transcriptional regulation of termite castes is therefore still unclear, especially in higher termites that form complex colonies with lifelong sterile worker castes. In this study, we aimed to fill this gap by analysing DNA methylation patterns in the fungus-farming higher termite *Macrotermes natalensis*.

As in eusocial Hymenoptera, termites are also characterized by extreme longevity among fertile castes, while sterile castes are short-lived, indicating an apparent absence of the fecundity–longevity trade-off attributed to non-social insects [15]. Higher termites exhibit extreme examples of this disparity in longevity and fecundity between castes [16]. In *M. natalensis*, for instance, which is the focus of the current study, sterile workers live only weeks, while kings and queens can live for over 20 years [17], with the highly fertile queen laying thousands of eggs per day [18]. Several important genes and pathways have been indicated as important for longevity and fecundity in termites and other eusocial insects, such as the nutrient-sensing pathways Insulin/insulin-like growth factor (IGF-1) signalling (IIS) and target of rapamycin (TOR) [16,19,20], telomerase [21], transposon defence [16,22], oxidative stress [16,20,23], DNA damage repair and mitochondrial functions [20,23] Further, recent studies have also presented evidence for the transcriptional regulation of specific gene co-expression modules associated with old but highly fertile queens in ants [24], bees [25] and termites [20,26,27]. However, the role of DNA methylation in this absence of the longevity–fecundity trade-off in eusocial insects is so far unknown.

In this study, we investigated caste- and age-specific DNA methylation profiles to make inferences on the regulation of genes important for the extreme longevity and high fecundity of reproductives in *M. natalensis*. This foraging, fungus-farming termite is characterized by large colonies and sterile workers. The mature *Macrotermes* queens are characterized by a hypertrophic abdomen, as well as several further metabolic and physiological differences compared to virgin queens (VQ), such as enlarged corpora allata [28], increased DNA content and major changes in insulin signalling and fat storage [20].

We carried out reduced representation bisulfite sequencing (RRBS) on four phenotypes—short-lived, sterile female workers (FW), young VQ, 20-year-old queens and 20-year-old kings—replicated across three independent colonies from this higher termite and related DNA methylation patterns to caste- and age-specific gene expression. This was performed on the fat body, since we recently showed the importance of this tissue for the long reproductive life of the reproductive termite castes [20].

## 2. Results and discussion

### 2.1. Reduced representation bisulfite sequencing is a robust method for determining genomic methylation patterns in termites

For each of the four phenotypes, FW, VQ, mature queens (MQ) and mature kings (MK), we aimed to produce reduced RRBS for three replicates from independent colonies. With the RRBS method methylation status of a reduced portion of the genome is targeted, in which CpGs are expected to be enriched, as has previously been successfully employed to investigate phenotypic plasticity in the jewel wasp, *Nasonia vitripennis* [29]. An accurate estimation of methylation levels relies heavily on an efficient conversion rate of unmethylated sites with the bisulfite treatment. To measure the erroneous, non-conversion rates, each sample was supplemented with a non-methylated lambda spike-in control (see methods). All samples included in this study had a non-conversion rate lower than 2%, which can be considered sufficiently high for our analyses [30] (electronic supplementary material, table S1). We generated between 32.1 M and 61.4 M bisulfite treated reads per sample (electronic supplementary material, table S1). These reads were mapped to the genome (mapping rate: 67.3–71.2%; electronic supplementary material, table S1) to quantify methylation levels, and for each sample only CpGs to which at least 5 reads mapped were included in analyses. We were able to quantify methylation levels (at least 5 reads) of 6.29 million CpG sites (19.1% of all genomic CpGs), which compares well to previous RRBS studies [29,31,32]. For each phenotype, most CpGs were sequenced for all three replicates, ranging from 2.8 M to 3.6 M CpGs per phenotype (electronic supplementary material, figure S1). In support for the reliability of the RRBS method, a large proportion of the CpGs (1.97 M, 31.3%) were sequenced consistently within all 12 samples (4 phenotypes × 3 replicates), which was by far the largest intersection of the 12 sets of sequenced CpGs (electronic supplementary material, figure S1E). All subsequent analyses are based on this subset of 1.97 M CpGs (electronic supplementary material, S1).

### 2.2. High gene body methylation

**Figure 1.**
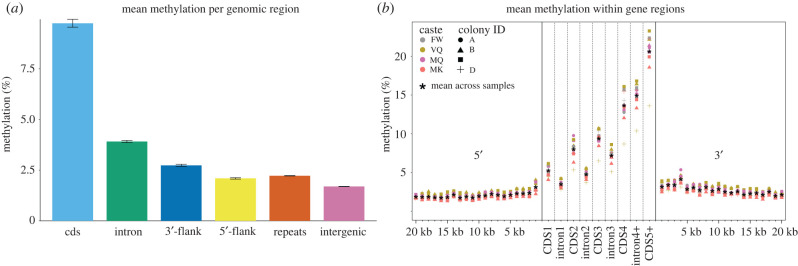
Genomic variation in methylation. In (*a*), mean methylation proportions among all 12 samples are shown for six categories of genome regions. Error bars are standard error. Flanks are defined as 10 kb up- or downstream of coding regions. (*b*) Mean methylation (%) within gene bodies (exons and introns) and in 20 1 kb bins at 5’- and 3’-flanking regions of genes. Each dot represents mean methylation for one of 12 samples across all sequenced CpGs within the region of interest. The four phenotypes are represented by colour; the colonies, from which replicates originated, are represented by shape. Asterisks show means across all 12 samples. FW, female workers; VQ, virgin queens; MQ, mature queens; MK, mature kings.

Within the subset of 1.97 M CpGs that were sequenced within all 12 individuals, we found detectable methylation at 49.0% (FDR corrected binomial *p*-value < 0.05, based on non-conversion rate) of sites in at least one sample. For each of the 12 samples, methylation level was calculated for each sequenced CpG as the proportion of mapped reads that were putatively methylated (non-converted cytosines). To estimate overall genomic methylation levels, we calculated means across the 12 samples at each CpG. Methylation levels differed significantly among genomic regions (*ad hoc* test: Kruskal–Wallis rank-sum test; *χ*^2^ = 23 761, *p* < 2.2 × 10^−16^, d.f. = 5; *post hoc* tests: pairwise Wilcoxon rank-sum test; all FDR-adjusted *p*-values < 4 × 10^−15^), with highest rates within coding regions (mean 9.74% per CpG, s.e.: 0.06) and lowest rates within intergenic regions (mean: 1.70%, s.e.: 2.75 × 10^−3^; figure 1*a*). In repetitive regions, methylation was higher than in intergenic regions (mean: 2.22%, s.e.: 3.8 × 10^−3^), indicating that TEs may be targeted by DNA methylation. Similar to findings for the lower termite, *Z. nevadensis* [12], methylation was relatively high in introns (mean: 3.91%, s.e.: 0.02; figure 1). In support of findings for *Z. nevadensis* [12] but in contrast to Hymenoptera [5,9], we found that, for all samples, methylation levels increased along the gene body, with highest levels at 3’ exons (13.6–23.3% among 5th to last exons) and introns (10.4–16.8% among 4th to last introns; figure 1*b*), suggesting this gene body methylation pattern may be widespread among termites.

### 2.3. Greater variation in methylation between colonies than between phenotypes

**Figure 2.**
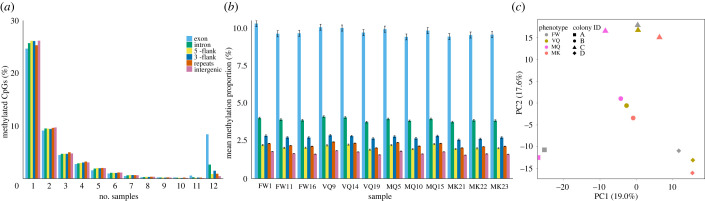
Individual variation in methylation. (*a*) Proportions of CpGs that are methylated (FDR < 0.05) in varying numbers of 12 samples within 6 genomic regions. (*b*) Mean proportions of methylated reads across all CpGs within six genomic regions for each of the 12 samples (4 phenotypes × 3 replicates). (*c*) Principal component analysis of methylation at 1000 most variable CpGs in 12 samples, spanning four phenotypes, represented by colour, from four colonies, represented by shape. The first two principal components are displayed on the *x*- and *y*-axes with variance explained in brackets. FW, female workers; VQ, virgin queens; MQ, mature queens; MK, mature kings.

As previously found for the clonal raider ant, *Oocera biroi* [8], we detected high individual variation in methylation patterns, with 24.7% to 26.2% of CpGs methylated in only 1 of the 12 samples, while only 9.1% to 9.7% were methylated in 2 individuals. Interestingly, as also found in *O. biroi* [8], we found a substantial number of CpGs (8.4%) within coding sequence and introns (2.7%) that were robustly methylated within all 12 samples (figure 2*a*). These robustly methylated CpGs were situated in genes enriched for GO-terms related to microtubule movement, protein phosphorylation, GTPase signalling and protein ubiquitination (electronic supplementary material, table S2). Interestingly, robustly methylated genes (containing at least one CpG methylated in all 12 samples) were more frequently differentially expressed between phenotypes (94.7%), compared to other genes (63.1%; *χ*^2^ = 8704, *p* < 2.2 × 10^−16^, d.f. = 1), suggesting an important role of DNA methylation in the regulation of gene transcription. Furthermore, methylation levels (proportion of mapped read at each CpG that were methylated), correlated strongly and similarly between all samples (Pearson’s *r*: 0.600–0.781; *p*-value < 2.2 × 10^−16^; electronic supplementary material, table S3), especially within coding sequence (0.889–0.960), indicating little differentiation between phenotypes, similar to findings for the subterranean termite, *R. speratus* [14]. The slightly lower correlations we report here compared to those found for *R. speratus* may be linked to a number of differences in this current study, such as colony replication, RRBS rather than whole-genome BS-sequencing, or may be related to species-specific patterns. Furthermore, the high correlations we found between VQ and MQ (0.663–0.778) suggest DNA methylation patterns are well maintained with age in termite queens. This apparent lack of epigenetic drift, at least for DNA methylation, may help to explain the recently documented, well-regulated transcription of anti-ageing genes in *M. natalensis* queens [20]. Previous studies have indicated the importance of low extrinsic mortality in postponing the selection shadow on reproductive castes, thus improving the efficacy of selection on longevity-related genes [16,33]. The high reproductive fitness in 20-year-old queens and the apparent lack of epigenetic drift we report here support the notion that these mature termite queens are not yet affected by a selection shadow.

Methylation levels also varied among individuals, with coding methylation ranging from mean 9.41% (±0.20 s.e.) in the mature queen from colony 5 (sample ID: MQ10) to 10.29% (±0.21 s.e.) in the female worker sample from colony 3 (FW1; figure 2*b*). Intergenic CpGs, on the other hand, were most highly methylated in the VQ sample from colony 5 (VQ9; mean: 1.85% ±0.01 s.e.) and lowest in the MK sample from colony 5 (MK21; mean: 1.56% ±0.01 s.e.). A principal component analysis revealed that methylation patterns vary more between colonies than between phenotypes (figure 2*c*), as previously found for the ants *O. biroi* [8] and *Dinoponera quadriceps*, and the paper wasp *Polistes canadensis* [9]. This highlights the importance of replication across independent colonies in methylation studies as previously reported [8], thus raising the question of whether caste-specific methylation patterns detected within a single colony for the lower termite *Z. nevadensis* were species- or colony-specific [12]. High colony variation is confirmed by Kruskal–Wallis tests among the 10 000 most variable sites, in which colony (*χ*^2^ = 2342.9, *p* < 2.2 × 10^−16^, d.f. = 3) has an effect size (generalized eta squared[ges] = 0.020) larger than that of phenotype (*χ*^2^ = 909.1, *p* < 2.2 × 10^−16^, d.f. = 3, ges = 0.008), while genomic region (exon, intron, 5’-flank, 3’-flank, repeats, intergenic) was an even stronger predictor of methylation level (*χ*^2^ = 3405.2, *p* < 2.2 × 10^−16^, d.f. = 5, ges = 0.028).

### 2.4. Conserved, single-copy genes are more highly methylated

**Figure 3.**
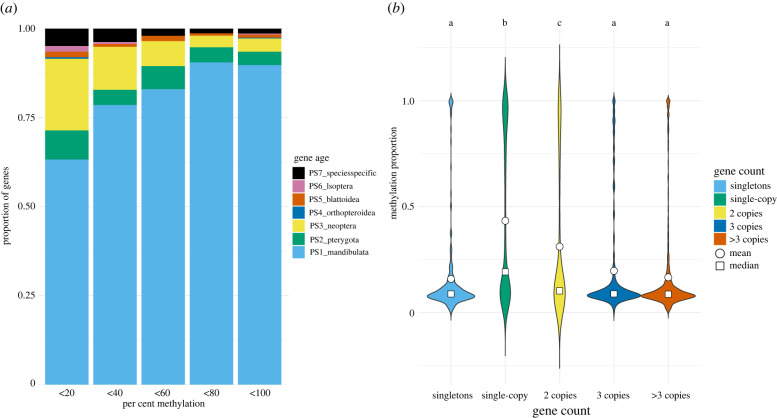
Methylation and gene conservation. (*a*) Proportions of gene age categories within five categories of methylation level. (*b*) Methylation level within genes with varying numbers of copies. Singleton = no paralogues or orthologues; single-copy = no paralogues but with orthologues in other species; other gene groups have varying numbers of paralogues.

We performed two analyses which confirmed higher methylation levels for conserved genes. We first analysed gene age by determining the broadest phylogenetic taxon for which a gene orthologue could be found, ranging from species-specific to Mandibulata. The proportion of highly conserved genes, found in the oldest category, Mandibulata, was highest among genes with methylation levels greater than 80%, while species-specific genes were proportionally most abundant among lowly methylated genes (figure 3*a*). In further support for greater methylation of conserved genes, we found significantly higher methylation levels among single-copy orthologue genes (single copy in *M. natalensis* with orthology in other insects) than in multi-copy genes (greater than 2 paralogues). Similarly, for singletons (single-copy, species-specific genes), which are likely evolutionarily novel compared to orthologues, methylation levels were lower than in single-copy orthologues and did not differ from multi-copy genes. The methylation of 2-copy genes were intermediate between single-copy and multi-copy genes (figure 3*b*).

### 2.5. Ageing and fertility genes hypomethylated in mature queens

**Figure 4.**
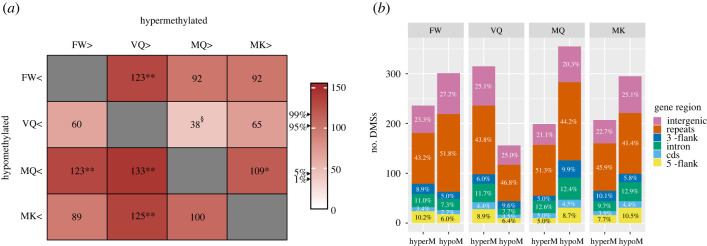
Differentially methylated sites. (*a*) Numbers of CpG sites hyper- (columns) and hypomethylated (rows) between pairs of phenotypes. Bootstrapping was carried out based on numbers of significant sites in 1000 comparisons between randomized 3 × 3 samples; 95% confidence interval: [45–102]; 99% confidence interval: [40–114]. ** >0.99; * >0.95; § <0.05. (*b*) Proportions of DMSs per genomic region for each phenotype. Unique DMSs were counted from all pairwise comparisons between the four phenotypes.

Despite the larger variation between colonies, we found 1291 CpG sites to be significantly differentially methylated sites (DMS) between phenotypes. We tested whether these numbers of DMS are greater or smaller than can be expected by chance by bootstrapping. For this, we randomly sampled from the 12 individuals, with replacement, two groups of three individuals and calculated the number of significantly DMS between the two groups. This was repeated 1000 times (1000 bootstraps; 95% confidence interval: [45–102]; 99% confidence interval: [40–114]). In this manner, we found a significant number of DMS that were hypermethylated in VQ compared to each of the other phenotypes (greater than 95%). In MQ, on the other hand, there were significant numbers of DMS that were hypomethylated compared to other phenotypes (greater than 95%; figure 4*a*). The numbers of unique DMS varied among phenotypes and genomic regions, and were enriched within coding regions, ranging from 2.7% hypomethylated in FW (*χ*^2^ = 4.5, FDR = 0.03, d.f. = 1) to 5.0% hypermethylated in MQ (*χ*^2^ = 22.2, FDR = 4.8 × 10^−6^, d.f. = 1), compared to the proportion of total sequenced CpGs within coding regions (1.2%). Interestingly, the largest category of DMS were those hypomethylated in MQ (365 unique sites) while the smallest category contained sites hypomethylated in VQ (163) (figure 4*b*). Of the 1291 DMS, 386 lay within 261 genes (DMGs), of which 111 genes contained sites hypomethylated in MQ, while 87 genes contained sites hypermethylated in VQ. These striking results indicate a major shift in methylation patterns occurs during queen maturation for a subset of genes.

Several of the genes with significantly decreased methylation in MQ compared to VQ have important roles in ageing, including 2 regulators of Notch signalling, 2 genes involved in Wnt signalling, a Sirtuin, a sphingomyelinase, important for cellular stress, and a gene responsible for the regulation of misfolded proteins (electronic supplementary material, table S4). Further genes are related to fertility such as Vitellogenin and an ecdysone receptor (electronic supplementary material, table S4). The role of the nutrient-sensing pathways, IIS and TOR, in the longevity and fecundity is well documented for social insects in general [16,19], as well as in *M. natalensis*, in particular, in which the major importance of non-conventional IIS in the fat body during the maturation process of queens has been highlighted [20]. It is therefore striking that *chico*, the substrate of insulin receptors in the IIS pathway, and *daw*, with known functions connected to the IIS pathway, are hypomethylated and differentially expressed (*chico* down-, *daw* upregulated) in MQ compared to VQ (electronic supplementary material, table S4). A large proportion of the 44 genes containing sites hypomethylated in MQ compared to VQ, were also differentially expressed: 6 were over-expressed in MQ (13.6%), 14 genes were lower expressed in MQ (31.8%) compared to VQ, while 24 (54.5%) did not differ in expression. These proportions of differentially expressed genes (DEGs) are significantly higher than those found in all genes (10.6% and 15.8%, respectively; *χ*^2^: 9.64, d.f. = 2, *p*-value = 0.008), indicating an important role of DNA methylation in the regulation of age-specific expression.

**Figure 5.**
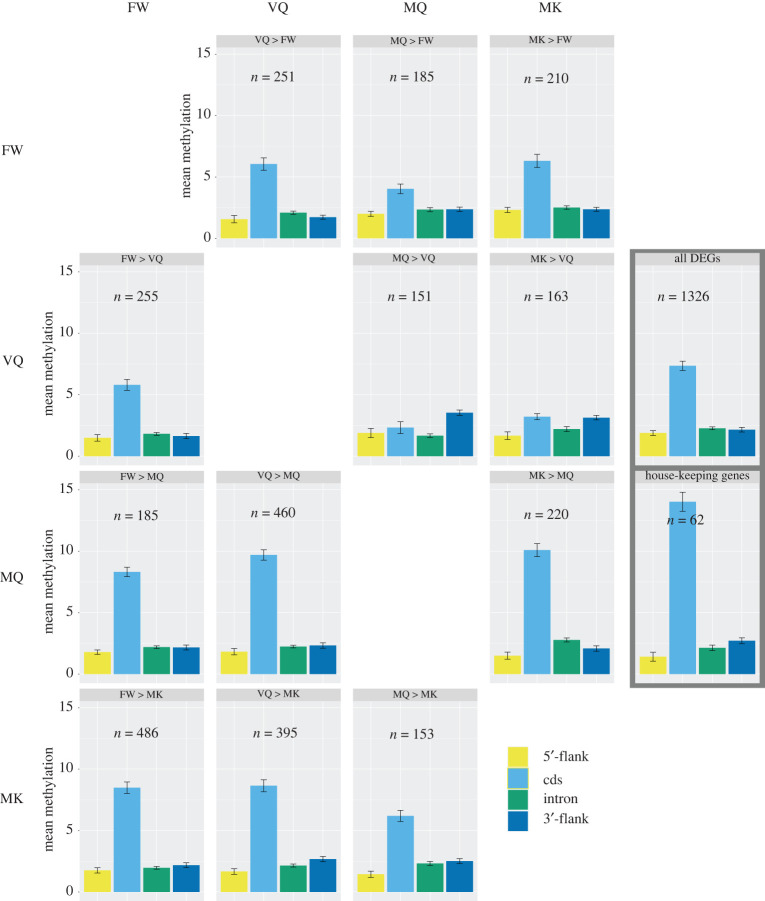
Mean methylation level per gene region for groups of differentially expressed genes. HKG, house-keeping genes, defined as non-differentially expressed genes, with expression counts greater than median expression. FW, female workers; VQ, virgin queens; MQ, mature queens; MK, mature kings.

Furthermore, we found that each category of DEGs (significantly upregulated or downregulated between pairs of phenotypes) had unique, phenotype-independent methylation signatures (figure 5). These patterns of methylation differed between groups of DEGs and within gene region (aligned ranks transformation ANOVA; *p* < 2.2 × 10^−16^). For instance, while the full set of DEGs have a mean methylation level of 7.4% in coding regions, genes with over-expression in MQ or MK compared to VQ, or in MQ versus FW, have very low coding region methylation (2.3%, 3.2% and 4.0%, respectively). Genes overexpressed in MQ and MK compared to VQ also have high methylation in 3’-flanks (3.5% and 3.1%, respectively), compared to all DEGs (2.2%) (figure 5). Surprisingly, within each of these DEG groups, variation among phenotypes was low, with standard deviation among samples ranging from 0.10 to 0.53. These patterns point towards a complex relationship between DNA methylation and caste- or age-specific gene expression in *M. natalensis*.

### 2.6. Variation in gene body methylation associated with expression level, caste-specific expression and alternative splicing

To better understand the variation in methylation levels among genes, we first investigated the influence of expression level. We found a significant positive correlation between methylation level of coding sites and expression level, which ranged from 0.208 (FDR = 2.0 × 10^−176^) to 0.254 (FDR = 8.2 × 10^−264^; Spearman’s rank correlation) per sample. This confirms previous findings for Hymenoptera [5,8,9] and a termite [12].

**Figure 6.**
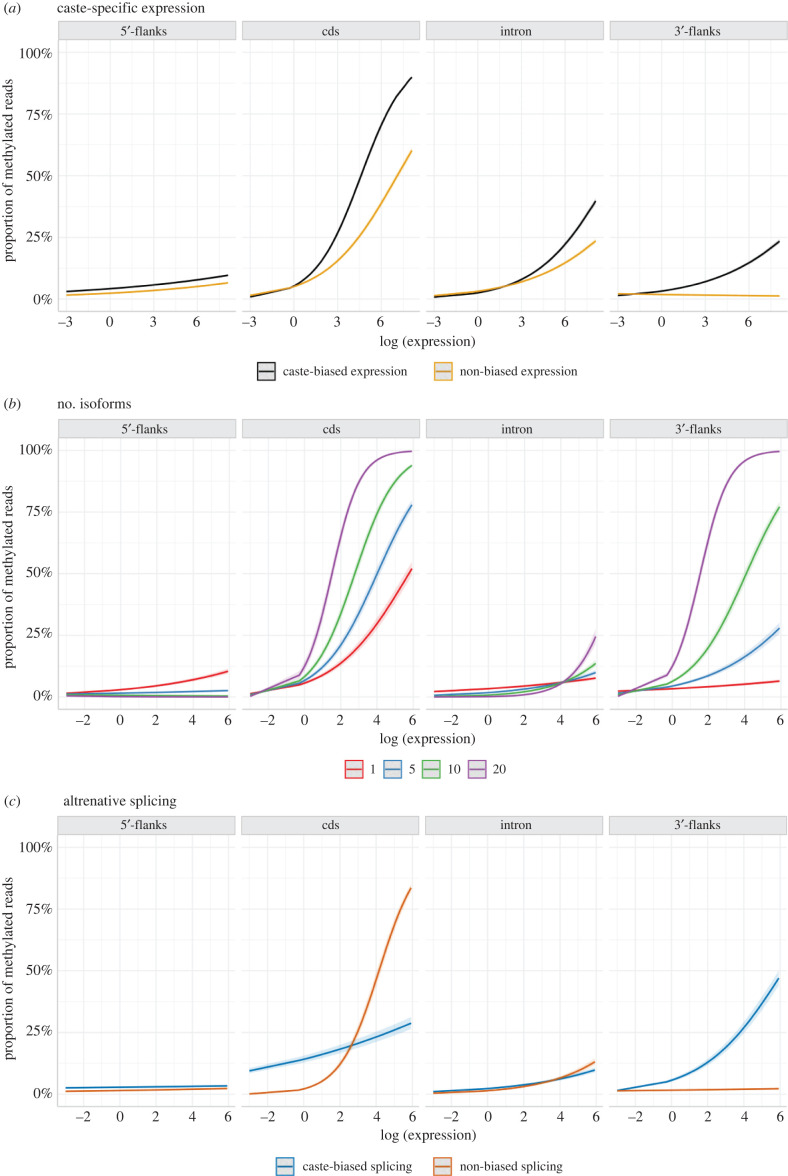
Generalized linear models with binomial distribution, relating gene expression level to methylation level depending on (*a*) differential gene expression, (*b*) number of isoforms and (*c*) caste-biased splicing, within coding regions, introns and 10 kb flanks. Models have the form: methylation level ∼ log (expression level) × variable × region + (1|sample).

Among genes whose expression differed significantly among phenotypes (DEGs), we found a significant positive interaction with expression, with a binomial regression predicting higher methylation especially in coding regions and 3’-flanks for DEGs compared to non-DEGs (figure 6*a*). A difference in methylation within introns between DEGs and non-DEGs was predicted only at high expression levels (greater than 67th percentile). We also found that methylation level increases in coding regions and 3’-flanks with the number of isoforms per gene, when controlling for expression level (figure 6*b*). For genes which are putatively differentially spliced among phenotypes (significant differential exon expression of multi-transcript genes), our regression predicts significantly higher methylation in 3’-flanks regardless of expression level and in coding regions at expression levels lower than the 83rd percentile (figure 6*c*). We found no difference in methylation levels within 5’-flanks. These results suggest an important role of DNA methylation in exons and 3’-flanks in the regulation of gene expression level, especially when regulating caste- and age-specific transcription and splicing. The regulation of caste-specific splicing via DNA methylation may be universal in eusocial insects since similar evidence has been found in honeybees [4], ants [5,8] and the lower termite, *Z. nevadensis* [12].

## 3. Conclusion

We report a strong correlation of DNA methylation patterns with caste- and age-specific gene expression and alternative splicing in the fat body of the higher termite *M. natalensis*. These results offer further support for the importance of fat body transcription [20] and its regulation for the extreme longevity and fecundity of termite queens. We also confirm the importance of replication in methylation analyses due to higher variation in methylation between colonies than between castes, a point of contention among previous studies in Hymenoptera [8]. Furthermore, and importantly, we present evidence for unique methylation signatures which are stable between phenotypes but differ especially between groups of genes with age-biased expression. For example, genes with higher expression in mature reproductives (MQ and MK) than in young reproductives (VQ) have relatively low coding region methylation but high methylation in 3’-flanks among all phenotypes compared to other DEGs. We believe this is the first time such a methylation pattern has been presented for social insects and suggests its generality should be tested on further species. We show for the first time, how DNA methylation may be responsible for regulating genes which are central to termite queens maintaining high fertility at extreme ages. For the 20-year-old, highly fertile queens, we present evidence for well-maintained DNA methylation, in support of an apparent lack of epigenetic drift, a well-established hallmark of ageing [3]. Several genes with important roles in ageing and fertility, on the other hand, contain sites with significantly reduced methylation levels in MQ compared to young, VQ, many of which have significantly different expression levels in old compared to young queens.

## 4. Methods

### 4.1. Sampling

Termites were collected in 2016 as part of the experiments described in Séité *et al.* [20] from field colonies belonging to the University of Pretoria, South Africa. Field colonies opened to collect animals had been followed for over 20 years by Jannette Mitchell in an experimental field of the University of Pretoria (coordinates in electronic supplementary material, table S8) [20]. All samples, female workers (FW), VQ, MQ and MK were sampled from colonies that were known to be at least 20 years old. The expected high fertility of the MQ was confirmed by (i) observing large egg clutches upon opening the colonies, (ii) large ovaries after dissection (see electronic supplementary material, figure S2) and (iii) high expression of vitellogenin in MQ compared to other phenotypes (electronic supplementary material, table S5), a well-established indicator of egg production [34]. All samples were exported at −80°C from South Africa to France. For more details, see Séité *et al.* [20].

### 4.2. DNA extractions and sequencing

Total genomic DNA from the 12 termite samples (female workers, young VQ, MQ and MK; see electronic supplementary material, table S1 and [20] for sampling) was extracted from fat body using DNeasy Blood and Tissue kit (Qiagen), including RNase A treatment (Qiagen), according to the manufacturer’s instructions. Library construction was performed using the Premium Reduced Representation Bisulfite Sequencing kit (Diagenode). Briefly, for each sample, 100 ng of genomic DNA were digested using MspI for 12 h at 37°C. DNA ends were repaired and Diagenode indexed adaptors were ligated to each end of the repaired DNA. Each ligated DNA was quantified by qPCR using the Kapa Library quantification kit (Kapabiosystems) on a LightCycler 480 (Roche Life Science) prior to pooling (4, 5 or 6 samples per pool). Each pool was subjected to bisulfite conversion and desalted. Optimal PCR cycle number was determined by qPCR (Kapa Library quantification kit, Kapabiosystems) before the final enrichment PCR. Once purified using magnetic beads (AMPure XP, Beckman Coulter), library pools were verified on Fragment Analyzer and precisely quantified by qPCR using the Kapa Library quantification kit (Kapabiosystems). Each pool was denatured, diluted and spiked with a 10% phiX Illumina library before clustering. Clustering and sequencing were performed in single read 100 nt, 1 lane per pool, according to the manufacturer’s instructions on a Hiseq2500 using Rapid V2 clustering and SBS reagents. Base calling was performed using the Real-Time Analysis Software and demultiplexing was performed using the bcl2fastq software, both from Illumina. Non-conversion rate of bisulfite treatment was estimated with a spike-in control, and only samples with a non-conversion rate lower than 5% were kept for further analysis.

### 4.3. Preparation of RRBS data

The RRBS reads were prepared by following the Bismark protocol [35]. This included adapter trimming with Trim Galore, v. 0.4.4_dev (https://github.com/FelixKrueger/TrimGalore) at default settings with the additional—rrbs argument. Subsequently, Bismark was used to analyse methylation states. The *M. natalensis* genome [36] was indexed using the bismark_genome_preparation command, then sequenced reads were mapped to the genome using bowtie2, v. 2.3.4.3 [37]. Otherwise, standard parameters were implemented for the Bismark pipeline.

### 4.4. Methylation analyses

We extracted methylation and read coverage information from the thus produced bam files with the bismark_methylation_extractor command, with the arguments—scaffolds and—bedGraph. We only considered sites to which at least 5 reads mapped. Based on the non-conversion rate of a spike-in control, a binomial test was carried out to confirm the significance of a measured proportion of non-converted, and therefore putatively methylated, reads, as previously performed by Glastad *et al.* [12]. *p*-values were FDR corrected, and only corrected *p*-values < 0.05 were deemed methylated, and were otherwise counted as non-methylated. Sequenced cytosines (greater than or equal to 5 reads) were annotated with gene features—exons, introns, 10 kb flanking regions, repetitive regions—based on information stored in two GFF files, containing protein coding [36] and repeat element annotations [38]. Ambiguously classified CpGs were removed and all thus far non-classified CpGs were classed as intergenic.

### 4.5. Principal component analysis

The PCA analysis was performed in R, v. 4.0.2 [39]. For each CpG site that was covered by at least 5 reads in all 12 samples, we measured variance in methylation among samples and selected the 1000 most variable sites. The PCA was computed on these top variable sites with the prcomp function and the first two PCs were plotted with ggplot2 [40].

### 4.6. Regression models

For each gene, average methylation level was calculated per feature type (exons, introns, 5’-flank and 3’-flank) and per sample. All regression analyses were performed on this dataset. The following variables were considered:

**Table:** 

methylation:	average proportion of methylated reads	continuous [0, 1]
expression:	normalized expression level, taken from [20]	continuous ≥0
feature:	genic region	categorical (exon, intron, 5’-flank, 3’-flank)
transcripts:	number of transcripts per locus	continuous, positive integers
colony:		categorical (A, B, C, D)
DE:	division of genes into DEG and non-DEG [20]	categorical (DE, non-DE)
AS:	whether gene has differenential exon expression between castes	categorical (AS, non-AS)

To relate expression to methylation level, dependent on DE, transcripts and AS, we carried out generalized linear models (GLM) with the glmer function from the lme4 package [41], using a binomial distribution and weighting methylation level by the total number of mapped reads. The 12 samples were used as random effect. Expression level was log transformed. We used the ggpredict function from the ggeffects package [42] for plotting. Each model had the format

$$\begin{array}{rl} & \mathrm{glmer}\;(\mathrm{methylation}\log(\text{expression}+0.001,\text{base}=10)\\ & \;\times \text{variable}+(1|\text{sample}),\text{family}=\text{binomial},\text{weights}\\ & \;=\text{TotalReads}). \end{array}$$

### 4.7. Detecting differential methylation

To detect significant differences in methylation between phenotypes, we used the R package methylKit, v. 1.11.1 [43]. We analysed differential methylation between all pairs of the four phenotypes (FW, VQ, MQ and MK) and for each of these comparisons only included CpGs, for which at least 10 reads existed for all size samples (3 replicates × 2 phenotypes). A difference in methylation was only considered significant if it were at least 25% points and with an adjusted *p*-value < 0.05. Each CpG, which was significant within any of these comparisons, was considered a DMS. To validate the numbers of DMS between pairs of phenotypes, we repeated this analysis for 1000 random pairings of three samples, sampled without replacement, and recorded the frequency of DMS in each case.

### 4.8. GO term enrichment of robustly methylated genes

We extracted the unique list of genes which contained CpGs methylated in all 12 samples (figure 2*a*). A GO-term enrichment test was performed on this list of genes with topGO (v. 2.34.075) [44], using the elim algorithm [45], which is recommended by the developers of topGO as it considers the topology of the GO graph thus reducing false positives. Node size was set to 5, Fisher exact tests were applied, and we only kept GO terms that matched with 2 genes at least and with a *p*-value < 0.05.

### 4.9. Alternative splicing

Alternative splicing was estimated for each gene by measuring differential exon expression with the package DEXseq [46]. This pipeline involves first formatting the gff and then extracting exon read counts from sam files. These sam files had been created in a previous study by mapping RNAseq reads to the *M. natalensis* genome [20]. The DEXseq pipeline was followed at default settings and for each of the four phenotypes compared to the other three phenotypes, we determined genes containing significantly differentially expressed exons (adjusted *p*-value < 0.05) relative to whole-gene expression. These genes were considered putatively alternatively spliced.

Additionally, we assembled a genome-guided transcriptome from RNAseq data (accessions: SAMN17088123-SAMN17088147) [20], using the new tuxedo protocol [47]. Raw reads were trimmed using Trimmomatic (v. 0.38) [48] with parameters TRAILING:25 LEADING:25 SLIDINGWINDOW:4:20 AVGQUAL:20 MINLEN:50. Only reads with both pairs after trimming were used for the further analysis. The trimmed RNAseq reads were mapped to the genome with Hisat2 (v. 2.1.0) [49] at default settings for each library. Individual transcriptomes were assembled and merged into one with StringTie (v. 1.3.4) [47]. Numbers of transcripts per annotated gene were then extracted from the resulting gff.

### 4.10. Differential expression

All data on gene expression levels and caste- and age-biased expression were obtained from Séité *et al.* [20].

## Data Availability

RRBS sequences have been deposited on NCBI, available under the accession PRJNA742659. Scripts and detailed methods are available from the GitHub repository: https://github.com/MCH74/Mnat_Methylation. The processed methylation data are available as electronic supplementary material [50].
